# Evaluation of the power deficit of elderly people during stair negotiation: Which joints should be assisted at least by an exoskeleton and with what amount?

**DOI:** 10.1017/wtc.2022.1

**Published:** 2022-03-25

**Authors:** Max Böhme, Felix Weiske, Jens Jäkel, Johannes Zentner, Maren Witt

**Affiliations:** 1 Faculty of Engineering, Leipzig University of Applied Sciences, Leipzig, Germany; 2 Faculty V—Mechanical Engineering and Transport Systems, Technical University Berlin, Berlin, Germany; 3 Department of Biomechanics, Faculty of Sport Science, University Leipzig, Leipzig, Germany

**Keywords:** Exoskeleton, knee joint, lower extremity, power deficit, stair climbing

## Abstract

Climbing stairs can become a daily obstacle for elderly people, and an exoskeleton can assist here. However, the exoskeletons that are designed to assist stair climbing are actuated in different ways. To find a minimal actuation configuration, we identify the assist phases by evaluating the power deficit of 11 healthy but weak elderly people (72.4 ± 2.1 years; 69–76 years; 1.67 ± 0.10 m; 74.88 ± 14.54 kg) compared to 13 younger people (24.0 ± 1.8 years; 22–28 years; 1.74 ± 0.10 m; 70.85 ± 11.91 kg) in a biomechanical study and discuss moment characteristics. Three-dimensional kinematics and ground reaction forces were collected, and kinematics, kinetics, and power characteristics of each subject for ascent and descent were calculated using inverse dynamics. Significant differences for power between both groups were assessed with statistical parametric mapping method using dynamic time warping. During ascent, the largest significant power deficit of the elderly subjects occurs in the single stance phase (SSP) during pull-up in the knee joint. During descent, significant mean power deficits of 0.2 and 0.8 W/kg for the highest deficit occur in the ankle joint in the beginning of the SSP and also in the knee joint in the same phase. Therefore, an exoskeleton should address the power deficit for knee extension (ascent: 1.0 ± 0.9 W/kg; descent: 0.3 ± 0.2 W/kg) and could assist the ankle during ascent and descent by an additional plantar flexion moment of 0.2 Nm/kg each.

## Introduction

Climbing stairs is one of the most demanding activities in the daily life of elderly people. For ascending and descending stairs, the biomechanical requirements on the body, such as applied joint moments, are higher compared to the horizontal gait (Ridgway et al., 2015). The fact that climbing stairs is a demanding task is also shown by the three-times higher oxygen consumption compared to level walking (Teh and Aziz, 2002). The natural decrease in muscle strength due to aging leads to a power deficit (Kalache and Kickbusch, 1997). An exoskeleton is, according to the Standard Terminology for Exoskeletons and Exosuits (ASTM F48 Committee, 2020), a wearable device that augments, enables, assists, and/or enhances physical activity through mechanical interaction with the body. It would be able to compensate the power deficit of elderly by addressing residual power and assisting movement directly.

### Biomechanics of Stair Climbing

Previous research on the biomechanics of stair climbing has shown that there are different ways of negotiating stairs: with the step-by-step or step-over-step method (Reid et al., 2007). In the step-by-step method, both feet are placed on the same step before ascending or descending. When climbing stairs using the traditional step-over-step method, only one foot is placed on each step alternately. This article focuses on the step-over-step method. In the past, the stair cycle was divided into stance and swing phases (SPs), with the mean transition at about 60% cycle time (Andriacchi et al., 1980). The stance phase can be further divided into the double stance phase (DSP) and single stance phase (SSP). Furthermore, subdivisions such as weight acceptance, pull up or controlled lowering, forward continuance, foot clearance, and foot placement were made for ascent and descent (McFadyen and Winter, 1988).

In general, the kinematics, moments, and power of stair climbing have been investigated in several studies in the past and are summarized in Tables A1 and A2 in Appendix A. The kinematics show a high variability of the hip joint angle curves in sagittal plane for ascent and descent (Novak and Brouwer, 2011). Subjects with pain in the patella femoral joint (Salsich et al., 2001) and stroke patients (Ridgway et al., 2015) have a changed gait pattern in this movement task. In Riener et al. (2002), it is reported that the applied joint moments are dependent of the stair inclination. According to the study by Afschrift et al. (2014), the hip extension, knee extension, knee flexion, and ankle plantar flexion are the joint movements with the highest task requirements for stair ascent. The task requirements are the external moments and powers that are imposed to the joints during the task execution. For both ascending and descending, the task requirements are higher compared to other activities of daily living such as sit-to-stand or stand-to-sit movements (Afschrift et al., 2014). However, the joints with a high task requirement do not necessarily have a need for assistance as well, since the decrease in muscle force must not occur in each joint equally (Grimmer et al., 2019), and thus the required muscle forces and moments for the movement task can be sufficient in specific joints even at an advanced age. The general statement according to Startzell et al. (2000) is that the descent is considered more challenging than the ascent, although for descent only the knee extension is highly demanding (Afschrift et al., 2014).

To quantify the joints that need assistance and the required amount, the kinetics of elderly during stair negotiation should be considered more in detail. Maximum forces and moments of elderly people decrease continuously with increasing age (Grimmer et al., 2019). Since a decrease in muscle mass and thus muscle strength occurs at the age of 50 and above (von Haehling et al., 2010), the required joint moments can only be achieved with increased effort compared to the task requirements based on the younger counterparts. To determine the difference in effort amplitudes, differing data lengths of subjects should be taken into account using appropriate time normalization techniques. Traditional findings such as Novak and Brouwer (2011) are based on linear time normalization and a multitude of trigger events. However, recent findings suggest that time normalization techniques (Weiske et al., 2021) and trigger events (Honert and Pataky, 2021) modify biomechanical results greatly. To quantify the power deficit between two groups appropriately, the use of dynamic time warping (DTW) for time normalization with statistical parametric mapping (SPM) for alpha level correction is therefore useful. DTW shifts data in time to make similar events, such as extremum points and falling and raising times, happen at the same normalized time (Tanawongsuwan and Bobick, 2001; Zhi-Qiang et al., 2019). Remaining differences will be due to differences in amplitude rather than due to temporal shifts. SPM ensures that the familywise error rate stays at the desired overall significance level (Friston et al., 1994; Robinson et al., 2015). This is important since time correlations present within data can overestimate significance of multiple tests across time, also known as multiple comparisons problem.

That elderly people generate lower ankle and knee moments is known from Reeves et al. (2009). The lower cadence in elderly is also caused by a reduced quadriceps force (Hurley et al., 1998). Furthermore, elderly people have problems with balance and coordination when climbing stairs (Verghese et al., 2008; Reeves et al., 2009). Twelve percent of all falls are caused by negotiating stairs or steps (Do et al., 2015). Moreland et al. (2004) recommend increased muscle strength to prevent falling.

### Exoskeletons for Stair-Climbing Assistance

In this article, assistance means that the human body is assisted by one or more external moments through an exoskeleton, where specific joints are actuated in at least one direction. Consequently, exoskeletons have the potential to provide both, training capabilities and functional compensation, to enhance human mobility (Grimmer et al., 2019). Furthermore, exoskeletons are characterized in their development by their requirements, whereby the movement that has to be assisted can significantly influence the system design, the actuator technology, sensor technology, and the movement control. Exoskeletons can either be designed for universal use and assist multiple movements, or they can assist only one specific movement. In order to increase the mobility and consequently the quality of life of elderly people and to enable them to live barrier-free even outside their homes, an exoskeleton should assist the most critical movements in everyday life, such as walking on inclined ground, climbing stairs, or walking over longer periods (Grimmer et al., 2019), as well as sit-to-stand and stand-to-sit movements (Pott et al., 2017). This article focuses only on exoskeletons for stair-climbing assistance. The exoskeletons that assist elderly people or people with weak muscles during stair climbing are summarized in Table B1 in Appendix B. A common feature of these exoskeletons is the active assistance of knee extension, whereby some systems also assist knee flexion or other joints. Therefore, the differences are about the additional active assistance of plantar flexion (Joudzadeh et al., 2018) and the assisted knee flexion (Angold et al., 2006; Chandrapal et al., 2013). The exoskeletons reported in Jang et al. (2016), Zhang et al. (2018), and Zhao et al. (2019b) and the exosuit from Zhao et al. (2019a) are suitable to assist stair ascent only. However, these systems also differ in the characteristics of the actuated joints. The Passive Knee-Assisting Exoskeleton (PKAExo) uses purely passive assistance (Li et al., 2018).

The reasons for the different actuation configurations are the different biomechanical assumptions. Joudzadeh et al. (2018) chose an assistance percentage of 50% based on the averages of the required joint powers reported in Riener et al. (2002). In our opinion, continuous assistance is less suitable because a user will rather accept the interaction with the exoskeleton. From the user perspective, we are searching for the least possible assistance in order to maintain self-activity. In addition, continuous assistance is less energy efficient and requires a larger energy supply unit, since energy is constantly consumed and potentials for energy recuperation are also not utilized. Although the hip joint is involved in the stair-climbing task, they concluded that there is no need of assistance, because the purpose of the hip joint mostly is to balance the torso (Joudzadeh et al., 2018, p. 77), whereas in Jang et al. (2016) the stair ascent is only assisted with additional hip extension and flexion moments. They generated the desired assistance moment profiles based on Riener et al. (2002) and improved them substantially through the empirical experiments (Jang et al., 2016, p. 5,660). In the case of the exosuit by Zhao et al. (2019a), a constant assisting moment is applied on the knee joint to help the wearer to overcome gravity. The assumed needed mean moment for knee joint extension is based on findings from Nadeau et al. (2003). However, Zhang et al. (2018) concluded from the same source that the active joints should be arranged in the hip joint of the frontal plane and knee joint of the sagittal plane, respectively. However, Novak and Brouwer (2011) have shown that elderly people can apply a higher moment for hip abduction during ascent and descent compared to young subjects. Since the joint moments applied by elderly people in the frontal plane are higher than those of young people for both movements, not only in the hip but also in the knee and ankle (Novak and Brouwer, 2011), and the absolute amounts are considerably lower in comparison to the sagittal plane, an assistance for joints in the frontal plane is less assumed.

Despite the large amount of scientific knowledge on negotiating stairs, the need for assistance of elderly people is rather unclear. This is also seen by the different actuation configurations of the exoskeletons, which have been specially designed for elderly people. Most exoskeleton developers focus on the joint moments and do not consider the power characteristics or the power deficit. However, the joint power provides information about the phases where mechanical energy has to be generated or absorbed. This is relevant for an actuator design and a specification of the energy supply, since recuperation potentials could then be considered. In addition, continuous assistance is used throughout the stair cycle and not only at the time in the cycle when assistance is really needed. To the best of the authors’ knowledge, there is no biomechanical study that investigates the power deficit of elderly people during stair climbing and takes into account the trigger events and the appropriate time normalization technique.

### Objective of the Study

The aim of this biomechanical study is to investigate the power deficit of healthy but weak elderly people compared to younger people. We want to know in which phases during ascending and descending stairs elderly people could need assistance through an exoskeleton. For this reason, we analyze the power deficit by applying suitable time normalization techniques and also discuss the moment characteristics in sagittal plane. Furthermore, the aim of this study is to obtain insights about the joints that need assistance and their assistance amount. For this purpose, experimentally determined joint moments are compared with available joint moments calculated from the literature, and suggestions are made for the joints that need assistance and for the amount of assistance that is needed. With this knowledge, an exoskeleton can be designed that actuates only the required movements or joints to assist elderly people during stair climbing.

### Structure of the Article

This article is organized as follows: The material and methods are described first. Afterward, the results of the ascending and descending movements are shown briefly with their moment characteristics and the calculated available moment peaks of the relevant joints. Power characteristics and power deficits are shown in the results section as well. Subsequently, we discuss the results and conclude them in the last section.

## Material and Methods

### Participants

The study included 13 young subjects (six males; seven females; 22–28 years) and 12 elderly (five males; seven females; 69–77 years), approved by the local ethics committee. According to their own statements, all subjects were healthy at the time of the trial and had no orthopedic limitations. Persons with artificial joint replacement were excluded from the examination.

### Setup and Procedure

The investigation was carried out on a purpose-designed staircase with standard dimensions (step height = 160 mm; tread length = 280 mm) according to DIN 18065 (Deutsches Institut für Normung e.V., 2020) and a resulting inclination angle of 30°. The staircase consisted of four steps, a stair landing at the upper end and a railing comprising three sides. A force plate (Kistler MiniDyn type 9119AA2, Winterthur, Switzerland) was used in the second step to record the step reaction forces.

A marker-based motion capture system consisting of 12 active infrared cameras distributed throughout the room was used for the kinematic recordings (Qualisys AB, Goteburg, Sweden). Based on the cast model (Cappozzo et al., 1995) and the addition of two markers on the shoulder, a total of 36 passive infrared markers were attached: In addition, eight markers were attached medially and laterally to the knee and ankle joint of both legs for static capture to determine the joint axes and segment lengths.

Before the test was conducted, two static recordings were taken of each subject in “neutral-zero position” (von Salis-Soglio, 2015). Body height, mass, and foot lengths were also measured. The test persons then carried out all ascents and descents barefoot, at a self-selected speed, step over step, and without the use of the hand railing. One recording included one ascent and one descent, starting from a standing position in front of the first step. Three recordings were collected per leg and per movement task (ascent and descent). This resulted in a total of 12 recordings per test person.

### Measurements and Calculations

The kinematics were recorded at 100 Hz and the kinetics at 500 Hz. The software Qualisys Track Manager (Qualisys AB, Goteburg, Sweden) was used for recording and synchronization.

Subsequently, a check for completeness and errors was carried out. Due to partial masking of markers by the test bed, gaps in the trajectories were created, which could be closed by interpolation during data preparation. Due to the exclusion of incomplete data series, a total of 124 ascents and 141 descents were analyzed. Care was taken to ensure that at least three movement cycles of each subject were complete and interpretable. The results of the oldest participant were not taken into account due to the use of the handrail. The results thus contained datasets of 13 young (24.0 ± 1.8 years; 22–28 years; 1.74 ± 0.10 m; 70.85 ± 11.91 kg) and 11 elderly subjects (72.4 ± 2.1 years; 69–76 years; 1.67 ± 0.10 m; 74.88 ± 14.54 kg).

The calculation of the joint moments was carried out using the inverse dynamic approach with version 7.3.0 of the AnyBody Modeling System software (AnyBody Technology A/S, Aalborg, Denmark) (Damsgaard et al., 2006). A body model of the AnyBody Managed Model Repository (version 1.6.6) without arms was generated for each test person (Lund et al., 2017), whereby the body parameters, such as segment lengths, mass, and body height, were taken from the respective measurement data listed in Table C1 and entered. The hip joint centers were estimated according to Bell et al. (1989). The length–mass scaling of the human model was carried out taking into account the fat content according to Rasmussen et al. (2005). The measured data were smoothed with a low-pass filter (second order, 5 Hz Butterworth) and kinematical optimized according to Andersen et al. (2010) to calculate the joint angle curves. Since the same measurement method was used for all markers, the expected uncertainty per marker is also the same. Consequently, all markers were weighted equally. Finally, the joint moments of hip, knee, and ankle were calculated using the polynomial criterion with power 3 (Rasmussen et al., 2001) and normalized to the body mass. Since elderly people rely more on their hip abductors and the majority of stair-climbing work is done in the sagittal plane (Novak and Brouwer, 2011), the frontal plane will not be discussed further in this paper.

For both ascent and descent, the beginning of a cycle was defined with the first foot contact on the force plate (0% cycle time) and the ending is the subsequent contact of the same foot (100% cycle time). The stance phase of the observed leg ends when the contact on the force plate is detached, at the same time the SP begins. The end of the SP was determined by the kinematic data. Initial foot contact and toe off from the contralateral limb were identified by an algorithm using the kinematic data. Thus, the definition of the DSP during stance phase with their transition from double to single stance (early stance) or single to double stance (late stance) of stair ascent and descent was enabled. Therefore, each cycle of both ascent and descent consists of a DSP (early stance), an SSP, another DSP (late stance), and an SP. Further subphases can be established according to Zachazewski et al. (1993). Ascending stairs consists of weight acceptance (only during early stance), pull-up and forward continuance (both during SSP), late stance, as well as foot clearance and foot placement (both during SP). Descending stairs also consists of weight acceptance (during early stance), forward continuance (during SSP), controlled lowering (during SSP until the end of the late stance), as well as leg pull through and foot placement (both during SP).

Subsequently, the available age-dependent moment peaks (amps) for extension and flexion of hip, knee, and ankle were calculated in groups for the elderly and the young subjects according to Harbo et al. (2012):(1)
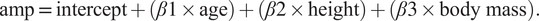


The intercept and the *β*-values were taken from the first table of Harbo et al. (2012). The age, height, and body mass from each subject (Table C1) were gender-specific averaged (young females: 22.6 years, 1.67 m, 61.5 kg; young males: 24.5 years, 1.83 m, 81.7 kg; elderly females: 70.7 years, 1.62 m, 70.2 kg; elderly males: 73.4 years, 1.74 m, 81.4 kg). Since the objective here was not to evaluate the results by gender, the calculated amps were averaged by group for the young and the elderly. The standard deviation (SD) of each amp was calculated as prediction interval with the values of Harbo et al. (2012) as well.

### Statistical Analysis

To calculate mean and maximum power deficits of elderly people, the power curves of both groups were compared. High similarities allow to summarize results from left and right legs (Novak and Brouwer, 2011) as independent samples each. User-specific gait patterns vary over several timing parameters like cadence or speed, while only the power deficit in amplitude during similar event times are necessary. To highlight differences in amplitude rather than differences due to shifted event times, we normalize time by DTW with one randomly drawn group sample each as reference signal (Honert and Pataky, 2021; Weiske et al., 2021). At each time step, a one-way Analysis of Variance (ANOVA) with SPM alpha-level correction identifies significantly different *F*-statistics (Friston et al., 1994; Friston, 2007; Robinson et al., 2015). SPM corrects the test at each time step to account for estimated time correlations present in the data with a target familywise error rate of 5%. Calculations were done within MATLAB 2020a (The Mathworks, Natick, MA, USA) using its Signal Processing Toolbox in combination with the spm1d package (Pataky, 2021).

Differences in the power curves of ankle, knee, and hip joints in the sagittal plane are calculated as mean deficit (mean young minus mean old) and highest deficit (mean + 1 SD young minus mean −1 SD old) for ascending and descending stairs. Significant power deficits occur when the proposed method identifies joint trajectories as significantly different between the young and the elderly. All curves are normalized to body weight.

## Results

**Figure D1.**
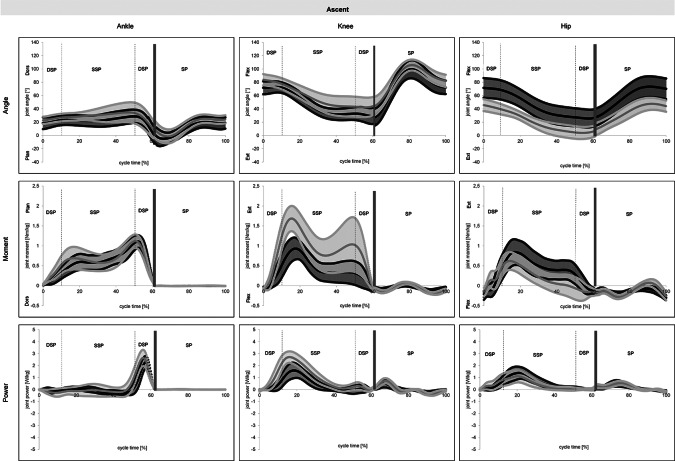
Mean values (±1 SD) of ankle, knee, and hip joint in the sagittal plane for young (gray) and elderly (black) subjects for stair ascent. Absolute cycle time young 1.40 ± 0.22 s and old 1.93 ± 0.37 s. Mean transition from stance phase to SP with 62.3 ± 17.1% of cycle time. *Abbreviations*: DSP, double stance phase; SP, swing phase; SSP, single stance phase.

**Figure D2.**
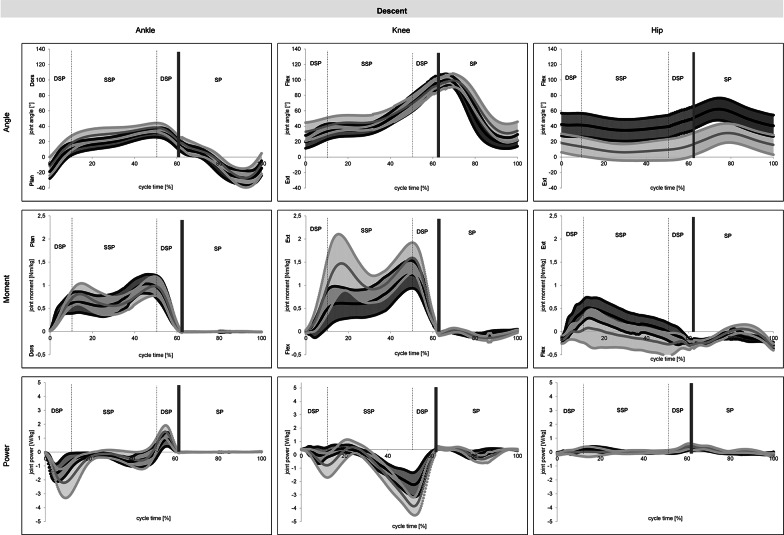
Mean values (±1 SD) of ankle, knee, and hip joint in the sagittal plane for young (gray) and elderly (black) subjects for stair descent. Absolute cycle time young 1.36 ± 0.32 s and old 1.87 ± 0.36 s. Mean transition from stance phase to SP with 62.3 ± 17.1% of cycle time. *Abbreviations*: DSP, double stance phase; SP, swing phase; SSP, single stance phase.

The angle, moment, and power characteristics are shown for the ankle, knee, and hip joint in Figure D1 in Appendix D for the ascent and in Figure D2 for the descent.

### Moment Characteristics and Available Moment Peaks

During ascent, two peaks each characterize the qualitative progression of the moments in the knee and ankle joints in the stance phase (Figure D1). In the hip joint, only one peak occurs in the SSP. The mean moment peaks (mmps) all occur in the stance phase and are shown in Table 1 for elderly and young subjects. During the SP, the mmp of the hip joint is 0.3 ± 0.1 Nm/kg for hip flexion. During SP, negligible moments occur in knee and ankle joint.

**Table 1.** Mean moment peaks (mmps) in Newton meter per kilogram with standard deviation (SD) for elderly and young people during ascending and descending stairs

	Ascent				Descent			
	Elderly		Young		Elderly		Young	
	mmp	SD	mmp	SD	mmp	SD	mmp	SD
Ankle plantar flexion	1.1	0.2	1.1	0.2	1.0	0.2	1.0	0.2
Knee extension	0.9^a^	0.3	1.7^a^	0.3	1.2^a^	0.3	1.6^a^	0.3
Hip extension	0.8^a^	0.3	0.6^a^	0.2	0.5^a^	0.2	0.1^a^	0.4
Hip flexion	0.3	0.1	0.3	0.1	0.3	0.1	0.3	0.1

a Significant differences between the elderly and the young according to one-way ANOVA *F*-Tests with *p* < .001.

During descent, two peaks each characterize the qualitative progression of the moments in the knee and ankle joints in the stance phase, during forward continuance, and at the end of the controlled lowering (Figure D2). In the hip joint, only one peak occurs in the beginning of the SSP. The mean maximum moments in Table 1 all occur in the stance phase. In the SP occur flexion moments with 0.3 ± 0.1 Nm/kg in hip joint. During SP, moments in knee and ankle joint are also negligible.

The results in Table 2 of the calculated available moment peaks according to Harbo et al. (2012) show that age has a significant influence. All six movements show lower maximum values for the elderly compared to the younger counterparts. Particularly significant are the decreases in the maximum extension moments of the knee with 1.0 ± 0.6 Nm/kg and the hip with 0.8 ± 0.9 Nm/kg.

**Table 2.** Available age-dependent moment peaks (amps) in Newton meter per kilogram with standard deviation (SD) according to Harbo et al. (2012). Predicted peak moment (Nm) = intercept + (*β*_1_ × age) + (*β*_2_ × height) + (*β*_3_ × body mass) with the values from Table C1 and the corresponding isokinetic values for intercept and *β* from Harbo et al. (2012)

	Elderly (72.4 years)		Young (24.0 years)	
	amp	SD	amp	SD
Ankle plantar flexion	1.1	0.5	1.6	0.5
Ankle dorsal flexion	0.3	0.1	0.4	0.1
Knee extension	1.6	0.6	2.6	0.6
Knee flexion	0.8	0.4	1.2	0.4
Hip extension	1.7	0.9	2.5	0.9
Hip flexion	1.3	0.5	1.8	0.5

### Power Characteristics and Power Deficit

Mean power peaks during ascent and descent are shown in Table 3 and occur all in stance phase. All mean power peaks of the elderly are lower or the same compared to the values of the young people except for the hip extension during the ascent.

**Table 3.** Mean power peaks (mmps) in watts per kilogram with standard deviation (SD) for elderly and young people during ascending and descending stairs

	Ascent				Descent			
	Elderly		Young		Elderly		Young	
	mpp	SD	mpp	SD	mpp	SD	mpp	SD
Ankle plantar flexion	2.3	0.5	2.6	0.7	1.5	0.6	2.2	1.1
Knee extension	1.6	0.6	2.7	0.5	2.6	0.9	4.2	0.7
Hip extension	1.4	0.5	1.1	0.5	0.3	0.1	0.4	0.2
Hip flexion	0.2	0.1	0.2	0.1	0.1	0.1	0.2	0.1

**Figure 1.**
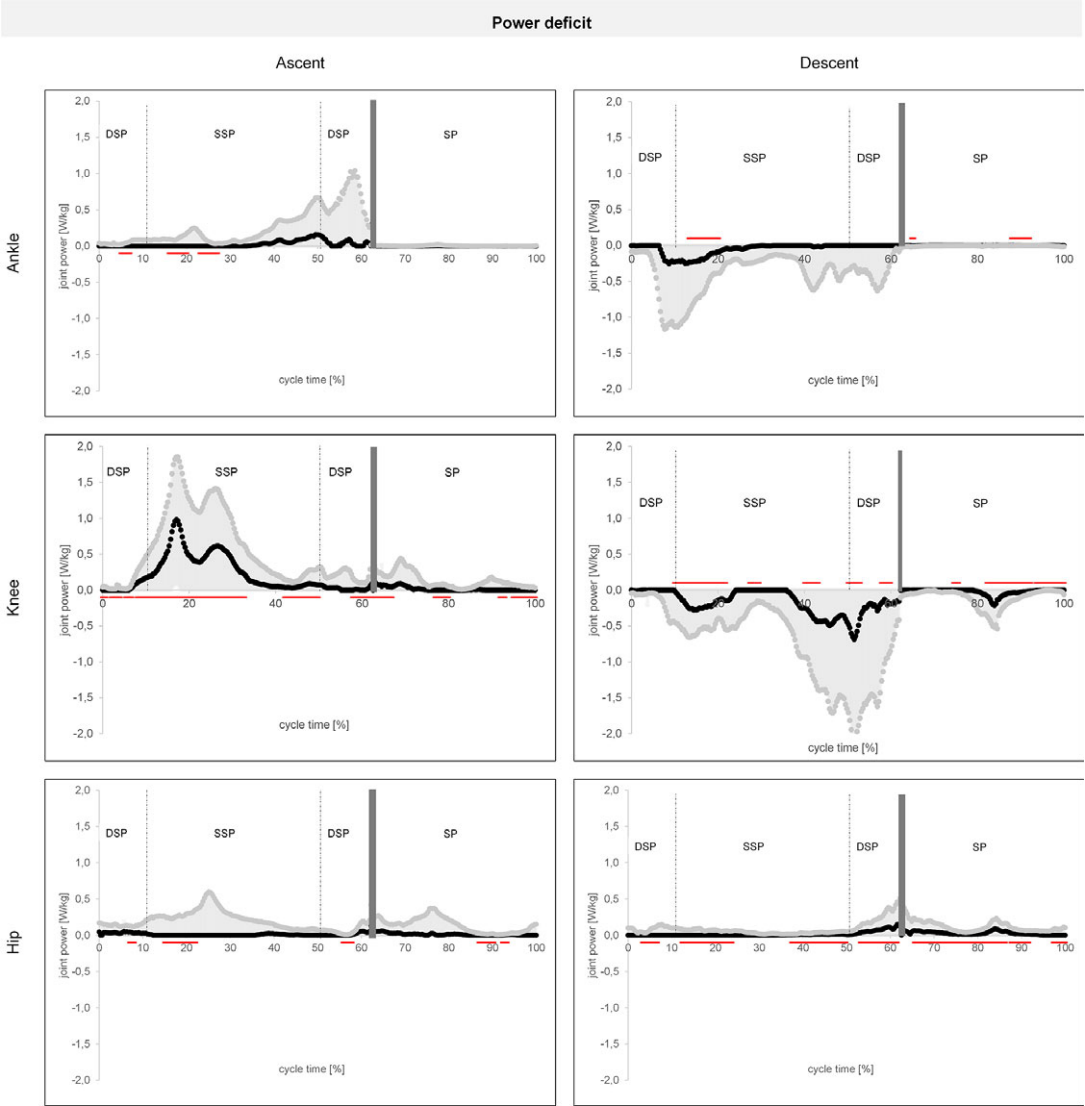
Differences in the power curves of ankle, knee, and hip joints in the sagittal plane as mean deficit (black; mean young minus mean old) and highest deficit (gray; mean + 1 SD young minus mean +1 SD old) for ascending (left column) and descending (right column) stairs. All curves are normalized to body weight. Red lines indicate the statistically significant sections of the corresponding joint trajectory comparisons by dynamic time warping and statistical parametric mapping (F). Transition from stance to SP at 62.3 ± 17.1% of the cycle time. Negative values represent eccentric muscle contractions, and positive values represent concentric muscle contractions. *Abbreviations*: DSP, double stance phase; SP, swing phase; SSP, single stance phase.

During ascent, the largest significant power deficit of elderly subjects is in the SSP during pull-up in knee joint with 1.0 ± 0.9 W/kg shown in Figure 1. At this phase, both the extension moment and the power in the knee joint are maximal. Other significant mean power deficits cannot be identified for the ascent. In the phase of pull-up at about 20% cycle time, the highest deficit is significantly higher in all three joints. In general, a significant power deficit across all three joints can be identified in the phase between 16 and 20% cycle time. All other significant phases of power deficit identified by the SPM show a negligible absolute amount.

During descent, as shown in Figure 1, two phases are significant across all three joints: from 13 to 20% during forward continuance and from 88 to 92% in the SP. During forward continuance in SSP, the absolute curves of the ankle joint show values of 0.2 W/kg for the mean deficit and 0.8 W/kg for the highest deficit. For the knee joint, mean deficits of 0.3 ± 0.3 W/kg can be identified. In the SP, the absolute values are negligible. Furthermore, individual phases during controlled lowering are significant in the knee joint, with values of 0.7 W/kg for the mean deficit and up to 2.1 W/kg for the highest deficit. In the hip joint, on the other hand, mean deficits of 0.2 and 0.5 W/kg for the highest deficit can be observed in the transition from stance to SP.

## Discussion

### Ascent

The results of the power deficit show that during the ascent in the phase of pull-up, the knee extension should be assisted in the peak at 16% cycle time with 1.0 W/kg mean (1.9 W/kg in the highest demand case). The joint moment curves from Figure D1 also show a much higher increase in the knee joint, which further indicates a need for assistance, as the required forces must be provided more quickly by the muscles compared to the ankle and hip joints. This increased deficit in the knee extension moment is contrasted by the age-related decrease in the maximum available moment. According to the results from Table 2, elderly people at mean age of 72.4 years have a maximum available moment of only 1.6 ± 0.6 Nm/kg, whereas the knee extension moment generated during ascent is already higher for the young counterparts with 1.7 ± 0.3 Nm/kg according to Table 1. It can therefore be concluded that the required joint moment for elderly people is 0.8 ± 0.6 Nm/kg, as they can only apply 0.9 ± 0.3 Nm/kg according to Table 1. Similarly, the absolute age-related loss of the maximum knee extension moment is the largest with a difference of 1.0 ± 0.6 Nm/kg compared to the other joints involved in ascending and descending stairs. The observed reduced knee joint peak in the elderly subjects shown in Figures D1 and D2 could be due to reduced quadriceps strength or other muscular decline. This is also consistent with the findings of Hurley et al. (1998), who identified reduced quadriceps strength as the cause of the lower cadence in elderly people.

For the ankle and hip joint in ascent, only small portions of the mean power deficit could be identified that are significant according to statistical evaluation (Figure 1). However, a power deficit cannot be concluded for the ankle and hip joint in ascent, because the amounts of the deficit in these two joints are negligible compared to the deficit in the knee joint. For the highest deficit in the significant phases according to Figure 1, there is only a value of 0.3 W/kg for the hip and ankle joint, which is also negligible compared to the power deficit in the knee. Furthermore, the maximum available moment according to Table 2 is 1.7 ± 0.9 Nm/kg for hip extension, whereas the maximum values according to Table 1 are 0.8 ± 0.3 Nm/kg for the elderly and even lower with 0.6 ± 0.2 Nm/kg for the young, so hip assistance can be ruled out. Since a redistribution of the moments toward the hip takes place, it can also be postulated that there is still sufficient muscle power available. However, a maximum available moment according to Table 2 for plantar flexion of only 1.1 ± 0.5 Nm/kg must be regarded as critical, since according to Table 1 maximum moments of 1.1 ± 0.2 Nm/kg are required here on the one hand, whereas, on the other hand, younger people can apply in the mean 0.5 Nm/kg more than required.

The exoskeletons described in Angold et al. (2006), Chandrapal et al. (2013), and Joudzadeh et al. (2018) all considered the identified power deficit in the knee joint. However, the exoskeletons of Angold et al. (2006) and Chandrapal et al. (2013) assist knee flexion, although there is certainly no power deficit after previous discussion. Only the exosuit described in Zhao et al. (2019a) assists the knee extension only. Since there is no power deficit in the hip, the exoskeletons by Jang et al. (2016), Zhang et al. (2018), and Zhao et al. (2019b) do not seem to be the ideal solution for assisting stair ascent. The passive system by Yuan et al. (2017) is also not able to prove sufficient torque at the knee for elderly people during ascent, because the maximum assisting moment through the exoskeleton is only 18 Nm and consequently much lower than the required 0.8 ± 0.3 Nm/kg.

### Descent

During descent in the beginning of the SSP between 13 and 20% cycle time, a mean deficit of 0.2 W/kg (0.8 W/kg as highest deficit) was identified in the ankle and 0.3 W/kg in the knee as mean (0.5 W/kg as highest) deficit in the peaks. The comparison of the required plantar flexion moment of 1.1 ± 0.2 Nm/kg according to Table 1 with the maximum available of 1.1 ± 0.5 Nm/kg from Table 2 is also critical, as in ascent. Therefore, the energy of the ankle should be absorbed in dorsiflexion or assisted by applying a plantar flexion moment of 0.2 Nm/kg on average (maximum 0.7 Nm/kg) according to the differences from Figure D2. In the knee, the energy of the flexion should be absorbed or an extension moment of 0.4 ± 0.3 Nm/kg should be applied. As power deficits of 0.7 W/kg in mean (maximum 2.0 W/kg) also occur in the knee joint during the phase of controlled lowering at 50% cycle time, an extension moment of 0.4 Nm/kg in mean (maximum 1.0 Nm/kg) according to the differences of the moment curves from Figure D2 should also be applied here to counteract flexion. Since the absolute power deficit in the SP is negligible, this should not be discussed further. For the hip joint, the descent shows parallels to the ascent: This involves the variability of the hip joint angle as already described in Novak and Brouwer (2011), the lower increase of the moments in the weight acceptance phase compared to the knee joint moment (Appendix D) and the negligible amounts of power deficit compared to the knee and ankle joint. The need for assistance for the descent is therefore correspondingly excluded for the hip, since the required moments of 0.5 ± 0.3 Nm/kg from Table 1 are also below the maximum available of 1.7 ± 0.9 Nm/kg.

Although it is known from the literature that the descent is more difficult than the ascent and that falls are more likely to occur here (Startzell et al., 2000), this cannot be clearly confirmed in the power deficit determined here. The increased risk of falls during descent is due to the degradation of vision, somatosensation, cardiovascular status, musculoskeletal health, cognition, and neurological status (Startzell et al., 2000). Therefore, the design of an exoskeleton should take into account not only the power deficit identified here, but also the nonmuscular limitations of elderly people mentioned above. Furthermore, factors of stair design, footwear, and clothing should be considered.

However, compensation of the occurring joint moment peaks in the knee and ankle joint should be considered for assisting during descent. With a suitable technical system, it would be possible to absorb energy with a spring or a damper from the phases of descent where negative work occurs, which could then be used to assist the ascent.

According to the results, the exoskeleton by Joudzadeh et al. (2018) is the only system that exclusively assists knee extension and plantar flexion. All other exoskeletons and exosuits assist more joints than necessary according to our findings, which is contrary to the strategy of minimal actuation. With a minimal actuator configuration, additional masses of the exoskeleton that affect the human body can be reduced to a minimum. However, it is also possible that the power deficit identified here is higher than the actual need, since it is not known how elderly would react in their motor control if only an adequate joint moment assistance is offered. This could only be evaluated afterward by developing a prototype and biomechanical tests, as it is described, for example, in Pott et al. (2017).

## Conclusion

In this study, we showed that a group of elderly people (and probably the majority of people in this age group) had a power deficit in the knee extension as well as in the plantar flexion of the ankle for both ascent and descent. Through the use of DTW with SPM, the differences in amplitudes of power curves were highlighted and the influence of shifted event times was reduced. Accordingly, the knee extension has to be assisted during ascent in the phase of pull-up with 1.0 W/kg in mean (maximum 1.9 W/kg) to relieve the knee joint extensor (quadriceps femoris). In descent, the knee should be assisted in the SSP during forward continuance and controlled lowering by applying an additional power of 0.3 W/kg (maximum 0.5 W/kg) at 13–20% cycle time and 0.7 W/kg (maximum 2.0 W/kg) at 50% cycle time, respectively, through the exoskeleton. The ankle could be assisted in ascent by an additional plantar flexion moment of 0.2 Nm/kg. In descent, a plantar flexion moment of 0.2 Nm/kg (maximum 0.7 Nm/kg) should be applied to assist the beginning of the SSP.

In general, the power deficit is very user-specific, as it depends on the subject’s physical condition. Elderly people can also be very fit at an advanced age, which is reflected in a nonexistent power deficit in sagittal plane. Further studies should also examine the frontal plane, since a need for assistance in this plane was not examined here and therefore cannot be completely ruled out. Nevertheless, with the assistance needs identified here, an exoskeleton can be developed that has a minimal actuator configuration and only provides the amount of required assistance at the appropriate times in the cycle.

## Data Availability

The data that support the findings of this study are available from the corresponding author, M.B., upon reasonable request.

## Appendix A: Stair-Climbing Results of Biomechanical Studies

**Table A1.** Stair ascent results in sagittal plane of other biomechanical studies supplemented by the results of this study. Presented are the mean values of the peaks from kinematics, moments, and power with flexion (F), extension (E), plantar flexion (PF), and dorsiflexion (DF)

Ascent																
Sources	Age in years: average (range)	Subjects	Cadence (steps/min.)	Kinematic peaks mean values (°)				Moment peaks mean values (Nm/kg)				Power peaks mean values (W/kg)				
				Hip	Knee	Ankle		Hip		Knee	Ankle	Hip		Knee	Ankle	
				F	F	PF	DF	F	E	E	PF	F	E	E	PF	DF
(Novak et al., 2011; Novak and Brouwer, 2011)	24 (20–30)	23	102	–	–	–	–	0.11	0.56	1.02	1.31	–	0.75	2.3	2.6	–
(Protopapadaki et al., 2007)	28 (18–39)	33 (16 m, 17 f)	–	65	94	31	11	0.76	–	0.58	–	–	–	–	–	–
(Reeves et al., 2009)	25	17 (10 m, 7 f)	98	–	94	9	23	–	–	1.19	1.48	–	–	–	–	–
(Reid et al., 2007)	24 (18–35)	17 (9 m, 8 f)	–	–	84	–	–	–	–	0.96	–	–	–	–	–	–
(Riener et al., 2002)	29 (24–34)	10 m	86	70	95	20	12	0.20	0.54	1.14	1.26	0.1	1.00	2.5	2.2	0.1
(Salsich et al., 2001)	32 (21–42)	10 (5 m, 5 f)	97	68	74	20	–	–	1.10	1.11	1.63	–	–	–	–	–
This study (young)	24 (22–28)	13 (6 m, 7f)	86	48	106	1	35	0.30	0.60	1.70	1.10	0.2	1.10	2.7	2.6	0.3
(Novak et al., 2011; Novak and Brouwer, 2011)	67 (55–83)	32	95	–	–	–	–	0.15	0.55	0.99	1.19	–	0.60	1.7	2.7	–
(Reeves et al., 2009)	75	15 (5 m, 10 f)	92	–	96	11	22	–	–	0.90	1.24	–	–	–	–	–
This study (elderly)	72 (69–77)	11 (6 m, 5 f)	62	73	100	5	29	0.30	0.80	0.90	1.10	0.2	1.40	1.6	2.3	0.3

**Table A2.** Stair descent results in sagittal plane of other biomechanical studies supplemented by the results of this study. Presented are the mean values of the peaks from kinematics, joint moments and power with F = flexion, E = extension, PF = plantar flexion, DF = dorsiflexion

Descent																
Sources	Age in years: average (range)	Subjects	Cadence (steps/min.)	Kinematic peaks mean values (°)				Moment peaks mean values (Nm/kg)				Power peaks mean values (W/kg)				
				Hip	Knee	Ankle		Hip		Knee	Ankle	Hip		Knee	Ankle	
				F	F	PF	DF	F	E	E	PF	F	E	E	PF	DF
(Novak et al., 2011; Novak and Brouwer, 2011)	24 (20–30)	23	111	–	–	–	–	0.39	0.23	1.06	1.07	–	0.30	3.5	1.9	2.2
(Protopapadaki et al., 2007)	28 (18–39)	33 (16 m, 17 f)	–	40	91	40	21	0.52	0.13	0.40	–	–	–	–	–	–
(Reeves et al., 2008)	25	17 (10 m, 7f)	94	–	92	21	33	–	–	0.91	1.32	–	–	–	–	–
(Reid et al., 2007)	24 (18–35)	17 (9 m, 8f)	–	–	83	–	–	–	–	0.50	–	–	–	–	–	–
(Riener et al., 2002)	29 (24–34)	10 m	100	43	95	20	18	0.60	0.00	1.35	1.12	0.2	0.40	4	1.4	2.4
(Salsich et al., 2001)	32 (21–42)	10 (5 m, 5 f)	86	26	92	28	–	–	0.53	0.78	1.47	–	–	–	–	–
Own study (young)	24 (22–28)	13 (6 m, 7f)	88	31	99	27	38	0.30	0.10	1.60	1.00	0.2	0.40	4.2	1.5	2.2
(Novak et al., 2011; Novak and Brouwer, 2011)	67 (55–83)	32	103	–	–	–	–	0.31	0.23	1.19	1.02	–	0.25	3.2	2.0	–
(Reeves et al., 2008)	75	15 (5 m, 10f)	–	–	90	23	34	–	–	0.83	1.00	–	–	–	–	–
Own study (elderly)	72 (69–77)	11 (6 m, 5f)	64	62	100	27	32	0.30	0.50	1.20	1.00	0.1	0.30	2.6	0.9	1.5

## Appendix B: Exoskeletons for Stair-Climbing Assistance

**Table B1.** Exoskeletons for stair-climbing assistance. User: people with weak muscles (WM), elderly people (EP), industrial worker (IW), needing rehabilitation (R). Assisted movement(s): stair ascent (SA), stair descent (SD), walking (W). Actuated joint(s): active (a), passive (p), hip (H), knee (K), foot (F), extension (e), flexion (f), adduction (add), abduction (abd), plantarflexion (pf), dorsal extension (de). Type of assistance: continuous (c), discontinuous (d)

System	Institution	Sources	Power supply	User	Assisted movement(s)	Actuated joint(s)	Type of assistance
Lower-Limb Exoskeleton (LLE) stair climbing	University of Canterbury	(Chandrapal et al., 2013)	Active	WM	SA/SD	a: K (e/f); p: F (pf/de)	d
LLE cable driven	University of Tehran	(Joudzadeh et al., 2018)	Active	EP	SA/SD	a: K (e); F (pf)	c
semipowered lower extremity exoskeleton	University of California (Berkeley)	(Angold et al., 2006)	Active	N/A	SA/SD	a: K (e/f)	N/A
Robotic hip exoskeleton	Samsung Advanced Institute of Technology	(Jang et al., 2016)	Active	EP	SA	a: H (e/f)	c
Hitexosuit	Harbin Institute of Technology	(Zhao et al., 2019a)	Active	EP	SA	a: K€	c
Lower extremity exoskeleton	Harbin Institute of Technology	(Zhang et al., 2018)	Active	EP, WM, and IW	SA (with increased load)	a: K (e/f); p: H (e/f)	c
Lower limb exo	Beihang University	(Zhao et al., 2019b)	Active	WM (R)	SA	a: H (e/f); K (e/f)	N/A
PKAExo	Chongqing University of Technology	(Yuan et al., 2017; Li et al., 2018)	Passive	N/A	W and SA	p: K(e)	c

## Appendix C: Anthropometric Data of the Test Persons

**Table C1.** Measured anthropometric data of the test persons with proband identification (PI), age (A) in years, gender (G [male (m), female (f)]), body mass (bm [kg]), body height (bh [m]), shank length (sl [m]), foot length (fl [m]), thigh perimeter (tp [m]), and shank perimeter (sp [m])

PI	A	G	bm	bh	Right side				Left side			
					sl	fl	tp	sp	sl	fl	tp	sp
1-01	26	m	76.2	1.76	0.400	0.290	0.570	0.390	0.391	0.295	0.580	0.390
1-02	25	m	81.2	1.83	0.422	0.280	0.620	0.400	0.419	0.280	0.600	0.390
1-03	28	m	88.0	1.93	0.463	0.295	0.560	0.395	0.454	0.295	0.565	0.395
1-04	22	f	69.1	1.64	0.367	0.240	0.575	0.385	0.365	0.240	0.570	0.380
1-05	23	f	67.9	1.63	0.361	0.245	0.550	0.365	0.360	0.245	0.550	0.365
1-06	25	f	60.7	1.63	0.368	0.240	0.560	0.340	0.360	0.245	0.555	0.340
1-07	23	f	65.3	1.73	0.390	0.250	0.540	0.360	0.385	0.250	0.540	0.360
1-08	25	f	60.8	1.66	0.378	0.225	0.535	0.350	0.371	0.230	0.510	0.350
1-09	23	f	55.5	1.67	0.358	0.235	0.470	0.350	0.357	0.240	0.480	0.340
1-10	22	f	51.5	1.72	0.397	0.235	0.465	0.325	0.385	0.235	0.470	0.320
1-11	23	m	79.4	1.78	0.384	0.265	0.550	0.370	0.385	0.265	0.550	0.370
1-12	22	m	88.3	1.82	0.423	0.275	0.530	0.400	0.424	0.280	0.530	0.400
1-13	25	m	77.1	1.87	0.436	0.275	0.540	0.370	0.421	0.275	0.550	0.375
2-01	69	f	92.6	1.75	0.427	0.260	0.630	0.400	0.418	0.260	0.610	0.385
2-02	72	m	81.1	1.77	0.402	0.270	0.490	0.340	0.398	0.270	0.520	0.350
2-03	73	f	77.2	1.55	0.355	0.240	0.530	0.385	0.348	0.240	0.530	0.370
2-04	73	m	70.9	1.66	0.372	0.260	0.490	0.355	0.365	0.260	0.490	0.360
2-05	74	m	99.6	1.78	0.428	0.295	0.494	0.388	0.419	0.300	0.510	0.387
2-06	76	f	42.1	1.49	0.321	0.225	0.440	0.290	0.317	0.225	0.440	0.290
2-07	69	f	69.7	1.62	0.375	0.240	0.560	0.380	0.369	0.240	0.550	0.390
2-08	74	f	69.4	1.76	0.419	0.270	0.510	0.400	0.411	0.270	0.505	0.390
2-09	72	f	62.6	1.59	0.368	0.235	0.500	0.340	0.362	0.230	0.500	0.340
2-10	72	m	73.7	1.71	0.388	0.260	0.470	0.350	0.383	0.260	0.480	0.350
2-11	77	m	81.7	1.76	0.418	0.290	0.470	0.360	0.418	0.290	0.480	0.360
2-12	72	f	78.0	1.58	0.371	0.250	0.510	0.370	0.370	0.250	0.500	0.380
